# Regional changes in thalamic shape and volume with increasing age

**DOI:** 10.1016/j.neuroimage.2012.07.043

**Published:** 2012-11-15

**Authors:** Emer J. Hughes, Jacqueline Bond, Patricia Svrckova, Antonis Makropoulos, Gareth Ball, David J. Sharp, A. David Edwards, Joeseph V. Hajnal, Serena J. Counsell

**Affiliations:** a Centre for the Developing Brain, Division of Imaging Sciences and Biomedical Engineering, King's College London, UK; b Imaging Sciences Department, MRC Clinical Sciences Centre, Hammersmith Hospital, Imperial College London, London W12 0NN, UK; c The Computational, Cognitive and Clinical Neuroimaging Laboratory, The Division of Experimental Medicine, Imperial College London, Hammersmith Hospital Campus, Du Cane Road, London W12 0NN, UK; d Imaging Physics and Engineering Group, Division of Imaging Sciences and Biomedical Engineering, King's College London, UK

**Keywords:** Healthy aging, Thalamus, Thalamo-cortical connectivity, Diffusion tensor imaging, Vertex analysis

## Abstract

The thalamus undergoes significant volume loss and microstructural change with increasing age. Alterations in thalamo-cortical connectivity may contribute to the decline in cognitive ability associated with aging. The aim of this study was to assess changes in thalamic shape and in the volume and diffusivity of thalamic regions parcellated by their connectivity to specific cortical regions in order to test the hypothesis *age related thalamic change primarily affects thalamic nuclei connecting to the frontal cortex*.

Using structural magnetic resonance imaging (MRI) and diffusion tensor imaging (DTI), we assessed thalamic volume and diffusivity in 86 healthy volunteers, median (range) age 44 (20–74) years. Regional thalamic micro and macro structural changes were assessed by segmenting the thalamus based on connectivity to the frontal, parietal, temporal and occipital cortices and determining the volumes and mean diffusivity of the thalamic projections.

Linear regression analysis was performed to test the relationship between increasing age and (i) normalised thalamic volume, (ii) whole thalamus diffusion measures, (iii) mean diffusivity (MD) of the thalamo-cortical projections, and (iv) volumes of the thalamo-cortical projections. We also assessed thalamic shape change using vertex analysis.

We observed a significant reduction in the volume and a significant increase in MD of the whole thalamus with increasing age. The volume of the thalamo-frontal projections decreased significantly with increasing age, however there was no significant relationship between the volumes of the thalamo-cortical projections to the parietal, temporal, and occipital cortex and age. Thalamic shape analysis showed that the greatest shape change was in the anterior thalamus, incorporating regions containing the anterior nucleus, the ventroanterior nucleus and the dorsomedial nucleus. To explore these results further we studied two additional groups of subjects (a younger and an older aged group, n = 20), which showed that the volume of the thalamo-frontal projections was correlated to executive functions scores, as assessed by the Stroop test. These data suggest that atrophy of the frontal thalamo-cortical unit may explain, at least in part, disorders of attention, working memory and executive function associated with increasing age.

## Introduction

Cognitive performance declines with age, particularly in the domains of processing speed, episodic memory and executive function (Cardenas et al., 2011; Kennedy et al., 2009). However, the specific neuro anatomical correlates of this cognitive decline remain unclear. The thalamus contains primary relay nuclei that have topographically organised projections to distinct zones of the cerebral cortex and plays a critical role in the coordination of information flow in the brain mediating communication and integrating many processes including memory, attention, and perception (Tuch et al., 2005; Ystad et al., 2010). Neuroimaging studies have demonstrated age related thalamic volume loss (Cherubini et al., 2009; Peran et al., 2009; Sullivan et al., 2004) and microstructural change (Abe et al., 2008; Cherubini et al., 2009; Ota et al., 2007). It is possible that micro- and macrostructural alterations in regions of the thalamus that are associated with cognitive performance contribute to age-related cognitive decline. This hypothesis can be tested by asking whether thalamo-frontal projections, which serve vulnerable processes like episodic memory and executive function, show greater age related changes than thalamic projections to other cortical regions.

Thalamo-cortical projections can be investigated in vivo by means of magnetic resonance imaging (MRI) including high resolution structural imaging and diffusion tensor imaging (DTI). DTI characterizes the diffusion properties of water molecules in tissue and can be used to assess the microstructural changes within the thalamus and provides the data for probabilistic tractography to classify thalamic grey matter by its connectivity to the cortex using connectivity based segmentation (Behrens et al., 2003). This method has been successfully used to reveal distinct sub-regions within the thalamus that correspond to histology (Johansen Berg et al., 2005).

In addition, detecting regional changes in thalamic shape facilitates investigations of normal and pathological variations in the brain. This approach has provided insight into regional thalamic degeneration in Alzheimer's disease (Zarei et al., 2010), Parkinson's disease (McKeown et al., 2008) and in schizophrenia (Coscia et al., 2009; Kang et al., 2008). In Alzheimer's disease, for example, the dorsomedial and intralaminar nuclei show the greatest reduction in volume compared to healthy controls (Zarei et al., 2010). Thalamic shape analysis may, therefore, provide further information on the effect of age on thalamo-cortical networks and aids comparison with the changes seen in mild cognitive impairment and Alzheimer's disease.

This study aimed to assess changes in thalamic shape and in the volume and diffusivity of thalamic regions parcellated by their connectivity to specific cortical regions in order to test the hypothesis that age related thalamic change primarily affects thalamic nuclei connecting to the frontal cortex.

## Methods

Imaging data for this study was taken from the IXI database, http://www.brain-development.org. Approval was granted by the Thames Valley Multicentre Research Ethics Committee and written informed consent was obtained from subjects prior to scanning. This study assessed data acquired at a single site, the Hammersmith Hospital.

### Subjects

Exclusion criteria were one or more of the following; left handedness, an abnormal radiological report including white matter lesions as assessed by a neuroradiologist on the conventional MR images, a history of neuropsychological illness or traumatic brain injury. Of the one hundred and forty nine subjects imaged at the Hammersmith Hospital, five subjects were excluded because they were left handed, fourteen were excluded due to an abnormal radiological report, twenty nine subjects had incomplete examinations or data that could not be retrieved from the archive, and seven were artefacted because motion and structural data from eight subjects were corrupt. This resulted in a study group of 86 subjects. The median (range) age of the subjects was 44 (20–74) years. Forty were male. There was no significant difference between the ages of males and females (p = 0.461). Seven subjects had raised cholesterol levels and four of these participants were receiving medication. Ten had high blood pressure and seven of these participants were also on medication. One participant was on medication for both high blood pressure and elevated cholesterol levels. All of these subjects were ≥ 60 years of age.

### Magnetic resonance imaging

MRI was performed on a Philips 3T system (Philips Medical Systems, Best, The Netherlands). 3D MPRAGE imaging (TR = 9.6 ms, TE = 4.5 ms, flip angle 8°, slice thickness = 1.2 mm, 0.94 mm × 0.94 mm in plane resolution, 150 slices) and dual echo (TR = 5205 ms, TE = 8 ms/100 ms, slice thickness = 2.4 mm, 0.95 mm × 0.95 mm in plane resolution, echo train length 16) were acquired. Single shot echo planar imaging DTI was acquired in 15 non-collinear directions (TR = 11,591 ms, TE = 51 ms, voxel size: 1.75 mm × 1.75 mm × 2 mm, 64 consecutive slices, b-value = 1000 s/mm^2^ and one image with no diffusion weighting, SENSE factor of 2).

### Image analysis

Image analysis was carried out using tools from FSL (FMRIB Software Library; http://www.fmrib.ox.ac.uk/fsl (Smith et al., 2004)).

#### Thalamic segmentation and vertex analysis

Automated segmentation of the thalamus was performed using FIRST, FMRIB's Integrated Registration and Segmentation Tool (Patenaude et al., 2011). The shape and appearance models used were constructed from manually segmented images provided by the Center for Morphometric Analysis, Massachusetts General Hospital, Boston. FIRST creates a surface mesh for each subcortical structure using a deformable mesh model. The mesh is composed of a set of triangles and the apex of adjoining triangles is called a vertex. The number of vertices for each structure is fixed so that corresponding vertices can be compared across individuals and between groups. Vertex correspondence is crucial for the FIRST methodology, as it facilitates the investigation of localised shape differences through the examination of group differences in the spatial location of each vertex. Although the vertices retain correspondence, the surfaces reside in the native image space, and thus have an arbitrary orientation/position. Therefore, the surfaces must all be aligned to a common space prior to investigating any group differences. The mean surface from the FIRST models (in MNI152 space) is used as the target to which surfaces from the individual subjects are aligned. The thalamus segmentation was performed by running a two-stage affine registration to standard space. The first stage is a 12 degree of freedom (DOF) registration to the non linear MNI152 template, and the second one is a 12 DOF registration using a subcortical mask, to exclude voxels outside the subcortical regions. Pose (rotation and translation) is removed by minimising the sum-of-squares difference between the corresponding vertices of a subject's surface and the mean surface (target) (Patenaude, 2007).

Vertex analysis was performed using first utils and thalamic shape change with increasing age was assessed on a per vertex basis. Regional changes in the vertices across all subjects were assessed using a global linear model with age as a continual regressor. The results were corrected for multiple comparisons using FDR (p < 0.05). The statistic was rendered on the shape surface, providing a map of the regions where the structure changed significantly with increasing age.

#### Thalamic volume

The 3D MPRAGE data were skull stripped using the brain extraction tool (BET) and affine-registered to MNI152 space (Jenkinson et al., 2001) using SIENAX in order to obtain a scaling factor for each subject's brain volume (Smith et al., 2002). The thalamic volumes were determined from the segmented thalami and were normalised for brain volume by multiplying thalamic volume by their scaling factor.

#### Parcellated thalamic diffusivity and volume measures using connectivity based thalamic parcellation

Image distortion due to eddy currents was minimised by affine registration of the diffusion images to the non diffusion image, b0. The DTI data were then skull stripped using BET and scalar maps of fractional anisotropy (FA), and mean diffusivity (MD), were generated by fitting a diffusion tensor model at each voxel using FMRIB's diffusion tool box (FDT).

Cortical masks (frontal, parietal, temporal and occipital cortices) were obtained from the Harvard Oxford subcortical and MNI structural atlas in FSL, were thresholded to exclude white matter and were then binarized. The segmented thalami were used as thalamic masks.

The DTI scalar maps were registered to standard space using a 2 step process as follows: 1. using FLIRT, each subject's b0 image was registered to its native 3D MPRAGE image, and 2. the 3D MPRAGE image was registered to the MNI_T1_1mm_brain using non-linear registration. The binarized thalamic and cortical masks were then propagated onto each individual's DTI scalar maps using the inverse of the above transformations. In order to exclude voxels that contained CSF along the medial border of the thalamus, the b0 images were segmented using FAST and a binarized cerebrospinal fluid (CSF) mask was used as an exclusion mask. Values for FA and MD were obtained for the whole thalami.

Thalamo-cortical connections were assessed using connectivity based seed classification incorporating the distance correction tool in probtrackX (Behrens et al., 2003). Each thalamic voxel was thresholded to include only those projections with a probability of ≥ 50% and binarized. Values of MD for each of the thalamo-cortical projections were determined. In addition, the volume of each projection was determined and normalised for brain volume. Previous studies have shown this approach to be highly reproducible (Traynor et al., 2010).

### Statistical analysis

Statistical analysis was performed using STATA (version 12.1). A simple linear regression analysis was performed to test the relationship between age and (i) normalised thalamic volume, (ii) whole thalamus FA and MD measures, (iii) MD of the thalamo-cortical projections, and (iv) normalised thalamo-cortical projection volumes. Scatter plots were created to illustrate the linear relationship between age and volume and age and MD in the thalamo-cortical projections.

Plots of residual measures against predicted measures found variances in some of the data that did not follow normal distributions and included some outliers. Since the assumption of normality of the data were not met in some cases, a further robust regression analysis, using a Cooke's distribution of > 1 to eliminate gross outliers was then performed. The change in MD and volume with increasing age in the thalamo-frontal projections was compared to all other thalamo-cortical projections using a Wald test in order to test whether the difference of the slopes of the regression (MD of thalamo-cortical projections versus age and volume of thalamo-cortical projections versus age) in each region differs significantly from zero. All results were corrected for multiple comparisons using a Bonferroni correction (0.05/22).

To investigate our results further we undertook a series of further exploratory and confirmatory studies. To explore the specificity of changes in frontal thalamo-cortical units we examined changes in regional cortical volumes with age. To confirm that the thalamo-cortical unit changed coherently with age we looked for allometric scaling between the frontal cortex and related thalamic projections. To assess the white matter components of the thalamo-cortical unit we measured FA and MD in thalamo-cortical tracts in each thalamo-cortical unit. To determine if changes in frontal structures were functionally relevant we undertook an additional study of a separate group of 20 subjects who underwent MR imaging and an assessment of cognitive function using the Stroop test.

### Confirmatory and explanatory studies

#### Assessment of cortical volume

The volume of the regional cortex masks was determined (see Parcellated thalamic diffusivity and volume measures using connectivity based thalamic parcellation) and the relationship between cortical volume and age was assessed using linear regression.

#### Allometric scaling in the frontal thalamocortical unit

Phylogenetic and ontogenetic studies have shown that thalamic nuclei and reciprocal cortical regions scale with a power law relation and an exponent of approximately 2/3 (Stevens, 2001; Kapellou et al., 2006). We tested whether the relationship between the frontal cortex and its reciprocal thalamic projection obeyed a power law relation consistent with this scaling law by determining the slope of the relationship between log frontal cortex volume and log volume of thalamo-frontal projection.

#### Fractional anisotropy and mean diffusivity in thalamo-cortical white matter tracts

Probabilistic tractography was performed using FSL (http://www.fmrib.ox.ac.uk/fsl) to assess FA and MD in thalamo-cortical white matter pathways. Pathways were generated using the thalamo-cortical projections as the seed masks and the reciprocal cortical regions as the waypoint mask (thalamo-cortical tracts, A), and vice versa (cortico-thalamic tracts, B). These tracts were normalised by the waytotal. Thalamo-cortical and cortico-thalamic tracts for each brain region were combined [(A + B) − (A ∗ B)] (Jbabdi. FSL support forum, https://www.jiscmail.ac.uk) and thresholded at a low level (0.01). The values for FA and MD of these tracts were extracted. The relationship between age and MD or FA was assessed using either a linear or quadratic fit as appropriate.

#### Assessment of thalmo-frontal connectivity and executive performance

We hypothesised that thalamo-frontal connectivity contributes to the decline in cognitive abilities associated with aging. In order to test this hypothesis we conducted a further study on 20 subjects, who were not part of the original study group, and addressed the question: *is the volume of the thalamo-frontal projection associated with scores of executive function?*

We studied 2 groups of 10 subjects; a younger aged group (median [range] = 25 [20–29] years) and an older aged group (median [range] = 71 [64–81] years). Exclusion criteria were as described in the Subjects section. Structural MR imaging was performed as described in the Magnetic resonance imaging section. DTI data were acquired in 64 non-collinear directions, with 4 b0 images and a b value of 1000 s/mm^2^. Data analysis was performed as described in the Parcellated thalamic diffusivity and volume measures using connectivity based thalamic parcellation section.

Executive function was assessed using the Stroop colour-word interference test (Stroop, 1935). The Stroop test consists of a reading condition in which the subject is required to read out loud as fast as possible the names of colour words; a similarly administered colour naming condition and an interference condition, in which colour naming is required of colour words printed in different coloured ink. The response to the word is slower and more error prone when the word is incongruent to the colour of the ink than when the meaning and the ink colour are congruent.

The Stroop test protocol was as follows: subjects were asked to name colour patches and were timed during the task (Condition 1), subjects were asked to read words that denote colours printed in black ink and were timed during the task (Condition 2). The average time taken to complete Condition 1 and Condition 2 was considered the Baseline. Subjects were asked to perform the interference task, where they are asked to read words that are printed in an incongruent coloured ink and were timed (Condition 3). The time taken to complete the baseline was subtracted from the time taken to complete Condition 3.

Scores for the Stroop test were tested for normality and found to be compatible with a normal distribution. Differences in Stroop test scores between the younger and older aged groups were assessed using a *t*-test. Linear regression analysis was used to assess the relationship between scores on the Stroop test and age. Robust regression analysis was performed to assess the relationship between scores for the Stroop test, and the volume of the thalamo-cortical projections. The data were corrected for multiple comparisons using a Bonferroni correction.

## Results

### Linear regression analysis of whole thalamus volume and DTI measures with increasing age

Thalamic volume decreased significantly with increasing age, (right thalamus, p < 0.001, R^2^ = 0.376, slope = − 37.752; left thalamus, p < 0.001, R^2^ = 0.307, slope = − 35.384). MD increased significantly with age (right thalamus, p < 0.001, R^2^ = 0.442, slope = 1.94 × 10^− 6^; left thalamus, p < 0.001, R^2^ = 0.2708, slope = 0.98 × 10^− 6^). There was no significant relationship between thalamic FA and age (right thalamus, p > 0.99, R^2^ = 0.003, slope = − 0.8 × 10^− 4^; left thalamus, p > 0.99, R^2^ = 0.032, slope = 0.2 × 10^− 3^).

### Thalamo-cortical connectivity

**Fig. 1:**
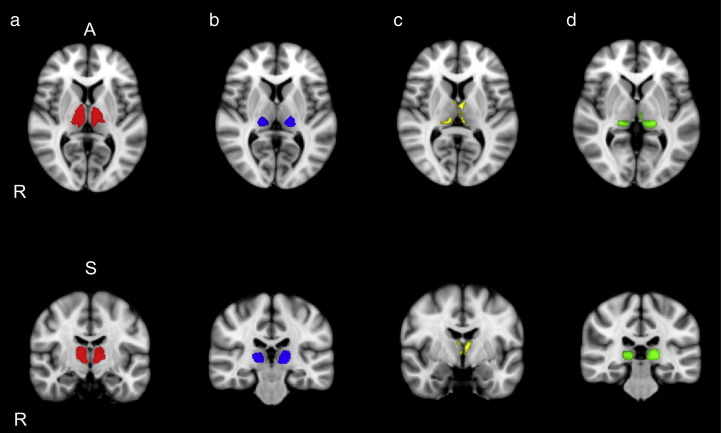
Mean thalamo cortical projections in MNI space. Columns a, b, c, and d show axial and coronal views of thalamic regions that were connected to the frontal cortex (red), parietal cortex (blue), temporal cortex (yellow) and occipital cortex (green) respectively.

The results of the thalamo-cortical connectivity analysis are shown in Fig. 1. Thalamo-frontal projections (red) included a large portion of the anterior part of the thalamus that covered the regions of the anterior nucleus, the ventroanterior nucleus and the dorsomedial nucleus. Thalamo-parietal projections (blue) included the posterolateral portion of the thalamus from the superior to the inferior border, incorporating the regions of the posterior ventrolateral nuclei and the ventroposterior nuclei. Thalamo-temporal projections (yellow) included the medial border of the thalamus from the anterior to posterior surface and incorporated parts of the lateral dorsal nucleus and medial aspect of the pulvinar. Thalamo-occipital projections (green) occupied the ventroposterior part of the thalamus including the regions of the pulvinar and lateral geniculate nuclei.

**Fig. 2:**
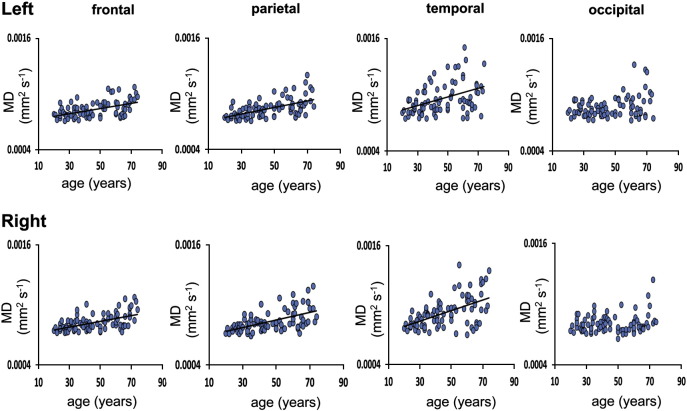
Linear regression plots for mean MD (mm^2^/s) of the left and right thalamo-frontal, thalamo-parietal, thalamo-temporal and thalamo-occipital projections with age. A regression line is shown for those regions where a significant robust linear regression with age was found (Bonferroni correction; p < 0.05/22).

As whole thalamic FA did not show a significant change with increasing age, only MD of the thalamo-cortical projections was assessed. Linear regression analysis showed that, with the exception of the thalamo-occipital projections, MD in all thalamo-cortical projections increased significantly with age (Fig. 2, Table 1). Age related change in MD in the thalamo-frontal projections was not significantly different to age related change of MD in the thalamo-cortical projections to other cortical regions (Left frontal versus left parietal, F = 2.88, DOF = 81, p = 0.2; left temporal, F = 0, DOF = 81, p = > 0.99; left occipital F = 2.26, DOF = 81, p = 0.41. Right frontal versus right parietal, F = 0.03, DOF = 81, p > 0.99; right temporal, F = 0.05, DOF = 81, p > 0.99 and right occipital, F = 3.6, DOF = 81, p = 0.1842). In order to determine whether these microstructural parameters were affected by the volume of the thalamo-cortical projections, this measure was included as a covariate in the regression model. Inclusion of the volume of the thalamo-cortical projections as a covariate did not change the results.

### Volumes of thalamo-cortical projections

**Fig. 3:**
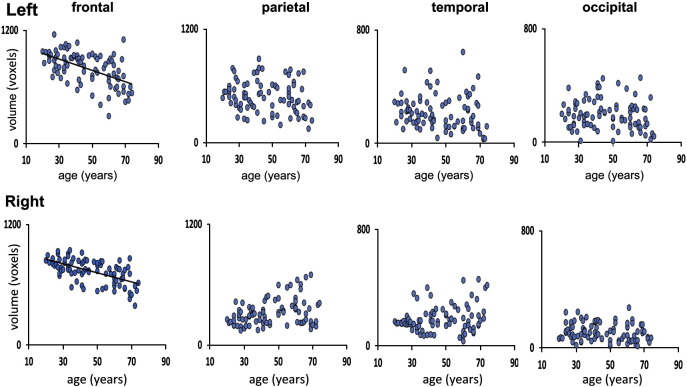
Linear regression plots for normalised volume (voxels) in the left and right thalamo-frontal, thalamo-parietal, thalamo-temporal and thalamo-occipital projections with age. A regression line is shown for those regions where a significant robust linear regression with age was found (Bonferroni correction; p < 0.05/22).

The volumes of the thalamo-frontal projections showed a significant negative linear relationship with age bilaterally. There was no significant relationship between the volumes of the thalamo-cortical projections to the parietal, temporal, and occipital cortex and age (Fig. 3, Table 2). Volume loss with increasing age in thalamo-frontal projections was significantly greater than volume loss in the thalamo-cortical projections to the parietal cortex (Left frontal versus left parietal, F = 8.83, DOF = 81, p = 0.012. Right frontal versus right parietal, F = 73.1, DOF = 81, P < 0.001), temporal cortex (Left frontal versus left temporal, F = 9.34, DOF = 81, p = 0.009. Right frontal versus right temporal cortex, F = 42.3, DOF = 81, p < 0.001), and left occipital cortex (Left frontal versus left occipital, F = 14.21, DOF = 81, p = 0.001). However, there was no difference in the age related volume loss in the right frontal projections when compared to the right occipital projections, F = 2.65, DOF = 81, p = 0.3219. Given this finding, and to further explore regional changes in thalamic volume with increasing age we assessed thalamic volume changes on a per vertex basis using shape analysis.

### Thalamic shape change with increasing age

**Fig. 4:**
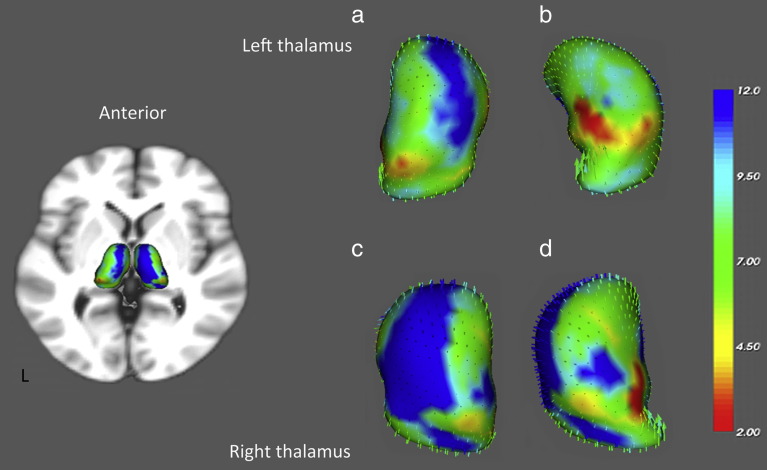
Main picture shows the results of the shape analysis for the left and right thalami in MNI space. All regions showed a significant correlational change in shape with age (F = 4.5–12), Blue represents the regions of the thalamus that showed the highest significant correlated changes in shape with age (F = 12). Red regions (F = 2) represent the areas where no significant correlation was found between changes in shape and age. Panels A and B show a magnified view of the shape analysis for the left thalamus, and panels C and D show magnified views for the right thalamus with vectors representing the direction of the shape change (most vector arrow heads cannot be visualised as they point inwards). B. Rotated to view the lateral surface of the left thalamus. D. rotated to view the lateral surface of the right thalamus.

The thalami showed significant age related changes in shape, suggesting atrophy (Fig. 4). The greatest shape changes with increasing age were observed in the anterior and medial aspects of the thalami bilaterally (Fig. 4). The lateral posteroventral thalamic surface did not show any significant change in shape with increasing age (Figs. 4b and d).

### Findings of confirmatory and explanatory studies

#### Assessment of cortical volume

The volume of the frontal cortex declined significantly with increasing age (p < 0.001 for both left and right frontal cortices), however there were no associations between age and volume in the other cortical regions. The change in frontal cortex volume was significantly greater than the change in the volume of the thalamo-frontal projection (p < 0.001), which led us to consider whether the changes in volume of the frontal cortex and thalamo-frontal projection might show allometric scaling.

#### Allometric scaling in the frontal thalamocortical unit

Plotting the relationship between log frontal cortex volume and log volume of thalamo-frontal projection generated a slope of 0.74 (CI 0.52–0.96) for the left thalamo-frontal unit and 0.79 (CI 0.51–1.08) for the right thalamo-cortical unit, consistent with previous estimates of mammalian thalamocortical scaling exponent. We explored this relationship further by introducing age and an appropriate interaction term into the model: this analysis confirmed the effect of age on cortical (p < 0.001 for left and right) and thalamic projection volumes (p < 0.001 for left and right) but showed no effect of age on the scaling exponent (left thalamo-cortical unit, p = 0.319; right thalamo-cortical unit, p = 0.904).

#### Fractional anisotropy and mean diffusivity in thalamo-cortical white matter tracts

FA values in the thalamo-frontal tracts decreased significantly with increasing age (left thalamo-frontal tract, p < 0.000, r^2^ = 0.168; right thalamo-frontal tract, p < 0.000, r^2^ = 0.191). FA values in all other thalamo-cortical tracts decreased with age but these relationships were no longer significant after Bonferonni correction (left thalamo-parietal, p > 0.99, r^2^ = 0.045; left thalamo-temporal, p = 0.064, r^2^ = 0.094; left thalamo-occipital, p > 0.99, r^2^ = 0.00; right thalamo-parietal, p > 0.99, r^2^ = 0.022; right thalamo-temporal, p > 0.99, r^2^ = 0.001; right thalamo-occipital, p > 0.99, r^2^ = 0.036).

MD increased significantly in the frontal and parietal thalamo-cortical tracts with increasing age (left thalamo-frontal tract, p < 0.0001; r^2^ = 0.283; right thalamo-frontal tract, p < 0.0001; r^2^ = 0.345; left thalamo-parietal tract, p < 0.0001; r^2^ = 0.196; right thalamo-parietal tract, p < 0.0001; r^2^ = 0.178). Increases in MD values in temporal and occipital thalamo-cortical tracts did not survive Bonferroni correction (left thalamo-temporal, p > 0.99, r^2^ = 0.046; right thalamo-temporal, p > 0.99, r^2^ = 0.012; left thalamo-occipital, p = 0.96, r^2^ = 0.065; right thalamo-occipital, p = 0.304, r^2^ = 0.092).

#### Assessment of thalmo-frontal connectivity and executive performance

Mean (± sd) Stroop test scores for the younger age group were 40.69 ± 10.46 s and for the older age group were 60.72 ± 12.72 s. Stroop test scores were significantly higher in the older aged group compared to the younger group (p = 0.001), reflecting an increased time to complete the task in the older aged group. Stroop test scores were significantly negatively associated with volume of the left thalamo-frontal projection (p < 0.001, r^2^ = 0.603) and right thalamo-frontal projection (p < 0.001, r^2^ = 0.535). There were no significant correlations between Stroop test scores and the volume of parietal, temporal or occipital thalamo-cortical projections (left thalamo-parietal projection, p = 0.215; left thalamo-temporal projection, p = 0.111; left thalamo-occipital projection, p = 0.237; right thalamo-parietal projection, p = 0.22; right thalamo-temporal projection, p = 0.141; right thalamo-occipital projection, p = 0.212).

### Discussion

This study explored alterations in thalamic shape and in the volume and diffusivity of thalamo-cortical projections with increasing age. Changes in thalamic shape were greatest in the anterior thalamus, which connects largely to the frontal cortex, and were minimal in the posterior ventrolateral region of the thalamus, which connects to the parietal cortex. These shape changes were mirrored in the volume of thalamo-cortical projections, with the greatest volume reduction being observed in thalamic regions connecting to frontal cortex and no significant changes in the volume of thalamo-cortical projections to other cortical regions.

The anterior thalamus, a region which includes the anterior nucleus and the dorsomedial nucleus, has connections with cortical and subcortical areas of the brain that play a crucial role in executive function, processing speed, and working memory, all of which are cognitive domains known to be affected by aging (Cardenas et al., 2011; Charlton et al., 2010; Grieve et al., 2007). The dorsomedial nucleus has reciprocal connections to the medial prefrontal, lateral prefrontal, and anterior cingulate cortices, a neural system that is involved with planning, problem solving, working memory (specifically in retrieval of episodic memory), mood and motivation (Channon, 2004; Smith and Jonides., 1999; Taber et al., 2004). The anterior nucleus has major reciprocal connections with anterior and posterior cingulate cortex and parts of the hippocampus via the fornix. This network is crucial for memory and directed attention (Bergfield et al., 2010; Giorgio et al., 2010; Taber et al., 2004). Degeneration of thalamic nuclei in these areas may, therefore, contribute to the cognitive decline that is associated with aging.

Our analysis of thalamo-cortical connectivity enabled us to explore diffusivity and volume changes in greater detail than at the level of the whole thalamus and the topography of our thalamo-cortical parcellations were similar to those described in previous studies (Behrens et al., 2003; Johansen-Berg et al., 2005). We found that, with the exception of the thalamo-occipital projections, mean diffusivity in all thalamo-cortical projections increased significantly with age. Our assessment of the white matter pathways connecting thalamus and cortex demonstrated that whilst MD increased in both thalamo-frontal and thalamo-parietal regions with increasing age, only reductions in thalamo-frontal FA with aging remained significant after multiple correction testing. These observed changes in the thalamo-frontal projections and thalamo-frontal white matter tracts occur in parallel with an age related decrease in frontal cortical volume and the volume of related thalamic regions. However, the relationship between the volume of frontal cortical and its thalamic projection remains constant throughout aging and is governed by a scaling law that has previously been described in primates (Stevens, 2001) and in infants (Kapellou et al., 2006).

Changes within the thalamo-cortical networks can have a significant effect on cognitive domains, such as working memory (Charlton et al., 2010), speeds of processing (Van Der Werf et al., 2001) and error awareness (Peterburs et al., 2011). Our data strongly suggest that aging effects operate not at the level of cortex but on the integrated thalamo-cortical unit, and the highly significant relationship between the volume of thalamo-frontal projections and executive function as assessed by the Stroop test (MacLeod, 1991), suggests that alterations in the integrity of the thalamo-frontal unit is an important factor in the cognitive decline associated with aging.

Our regional thalamic and cortical volume findings are supported by previous studies demonstrating that frontal lobe volumes show the greatest atrophy with increasing age, with relatively modest volume reductions in the parietal and occipital lobes (DeCarli et al., 2005; Jernigan et al., 2001). Of interest, in our present study, regions incorporating the ventrolateral nucleus and the ventroposterior nucleus showed relative preservation in volume on both thalamo-cortical projection and thalamic shape analysis. These regions project to primary motor and somatosensory cortices. As diffusivity in thalamo-parietal projections increases significantly with increasing age, these data suggest that changes in thalamic microstructure are not mirrored by macrostructural changes in all nuclei. Differences in the cytoarchitecture of the thalamus from region to region may also contribute to the regional differences of volume loss in the thalamus and highlights the benefit of using multi modal imaging for the assessment of thalamic degeneration in healthy aging.

A previous study observed that diffusivity in the anteriomedial thalamus did not change with increasing age (Ota et al., 2007). The reasons for these differing results are not clear. It may be because Ota et al. assessed a smaller subject group (28 subjects) or they may be due to differences in analysis approaches. The study by Ota obtained DTI in only 6 directions of diffusion sensitization and used manually drawn regions of interest to delineate the thalamus into areas with specific connections to the cortex without an intra-thalamic marker. As image contrast on conventional T1, T2 or diffusion imaging does not enable specific thalamic nuclei to be differentiated with confidence, we chose to explore thalamo-cortical connectivity using connectivity based segmentation (Behrens et al., 2003), which enables the thalamus to be segmented according to the probability of connection to regions of the cortex. This approach has been successfully used to segment the thalamus and has been found to correlate well with histological studies (Johansen Berg et al., 2005).

In accordance with previous studies (Cherubini et al., 2009; Peran et al., 2009), we demonstrated decreases in volume and increases in mean diffusivity with little change in FA values in the whole thalamus with increasing age. The underlying neurobiology of both the increased diffusivity and reduced volume may be due to the degeneration of neuronal structures including neuronal shrinkage, reductions of synaptic spines and reduced numbers of synapses (Terry et al., 1987). Recent positron emission tomography studies have shown an increase in activated microglia in healthy aging, suggestive of neuronal damage (Schuitemaker et al., 2012). However, whether this increase in activated microglia is related to neurodegeneration or is a direct reflection of the aging process on microglia is not clear (Conde and Streit, 2006).

A limitation of our study is the use of cross-sectional data to assess age related changes, which introduces potential confounding cohort effects, and so caution should be taken in extrapolating from cross-sections to longitudinal relationships. However, longitudinal studies which could confirm whether similar aging patterns exist in individual subjects are impractical over the wide age range assessed in this study (Cherubini et al., 2009).

In summary, thalamic shape and volume changes associated with healthy aging are heterogeneous, with anterior thalamic regions incorporating the anterior and dorsomedial nuclei, being most markedly affected. This selective vulnerability of the anterior thalamus to age related atrophy may explain, at least in part, disorders of attention, working memory and executive function associated with increasing age.

## Figures and Tables

**Supplementary Fig. 1:**
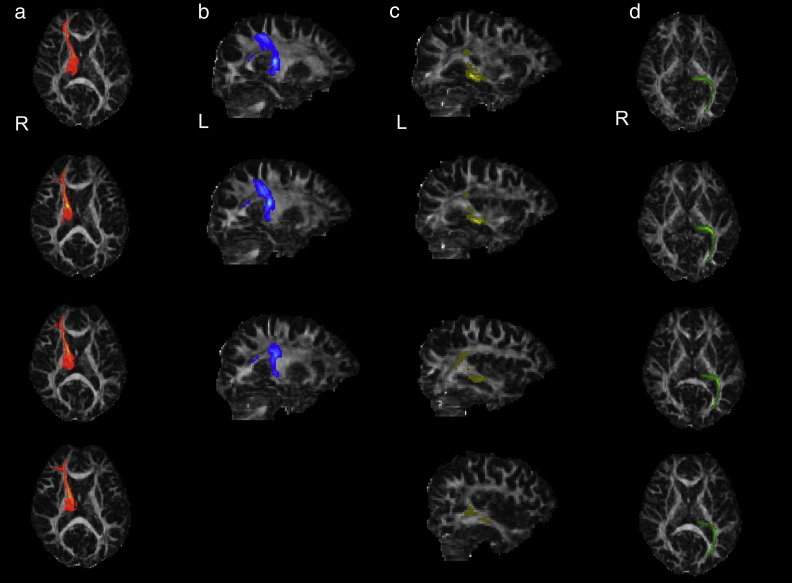
Thalamo-cortical tracts in a 55 year old subject. a. Thalamo-frontal tracts shown in the axial plane b. Thalamo-parietal tracts shown in the sagittal plane c. Thalamo-temporal tracts shown in the sagittal plane d. Thalamo-occipital tracts shown in the axial plane.

**Table 1:** Results of linear and robust regression analysis of MD of the thalamo-cortical projections with increasing age. Bold represents the analyses that survived Bonferroni correction.

		Linear regression of age versus mean diffusivity of thalamo cortical projections	Robust regression of age versus mean diffusivity of thalamo-cortical projections
Left thalamus	Frontal	**R² = 0.24, F = 27.1 slope = 2.74 × 10**^**− 6**^**, p < 0.001**	**F(84) = 24.57, p < 0.001**
Parietal	**R² = 0.39, F = 39.489, slope = 3.56 × 10**^**− 6**^**, p < 0.001**	**F(84) = 30.89, p < 0.001**
Temporal	**R² = 0.17, F = 16.761** **slope = 4.54 × 10**^**− 6**^**, p < 0.001**	**F(84) = 10.55, p = 0.002**
Occipital	R² = 0.0939,F = 8.705 slope = 2.176 **×** 10^− 6^, p = 0.083	F(84) = 2.32, p = 0.131
Right thalamus	Frontal	**R² = 0.295, F = 35.098, slope = 2.8 × 10**^**− 6**^**, p < 0.001**	**F(84) = 31.43, p < 0.001**
Parietal	**R² = 0.32, F = 39.33** **slope = 4.1 × 10**^**− 6**^**, p < 0.001**	**F(84) = 31.28, p < 0.001**
Temporal	**R² = 0.28, F = 34.675** **slope = 5.3 × 10**^**− 6**^**, p < 0.001**	**F(84) = 39.04, p < 0.001**
Occipital	R² = 0.183, F = 2.874 slope = 1.046 **×** 10^− 6^, p > 0.99	F(84) = 0.06, p = 0.813

**Table 2:** Results of linear and robust regression analysis of volumes of the thalamo-cortical projections with increasing age. Bold represents the analyses that survived Bonferroni correction.

		Linear regression of age versus volumes of thalamo cortical projections	Robust regression of age versus volumes of thalamo cortical projections
Left thalamus	Frontal	**R**^**2**^ **= 0.295, F = 35.185** **slope = − 5.99, p < 0.001**	**F(84) = 36.44, p < 0.001**
Parietal	R^2^ = 0.044, F = 3.878 slope = − 2.2239, p > 0.99	F(84) = 3.65, p > 0.99
Temporal	R^2^ = 0.025, F = 2.129 slope = − 1.063, p > 0.99	F(84) = 4.92, p = 0.642
Occipital	R^2^ = 0.014, F = 1.208 slope = − 1.0629, p > 0.99	F (84) = 1.47, p > 0.99
Right thalamus	Frontal	**R**^**2**^ **= 0.355 F = 46.17** **slope = − 4.522, p < 0.001**	**F(84) = 42.43, p < 0.001**
Parietal	R^2^ = 0.103, F = 9.693 slope = 2.6, p = 0.06	F(84) = 6.26, p = 0.314
Temporal	R^2^ = 0.052, F = 4.625 slope = 1.301, p = 0.748	F(84) = 1.38, p > 0.99
Occipital	R^2^ = 0.027, F = 2.28 slope = − 0.609, p > 0.99	F(84) = 2.08, p > 0.99
